# Single- and Double-Chain Arginine-Derived Surfactants: Antimicrobial, Antibiofilm and Synergistic Activities

**DOI:** 10.3390/ijms27135936

**Published:** 2026-07-01

**Authors:** Rafaela Gomes Bezerra, Lourdes Pérez, Zakaria Hafidi, Francisco Fábio Oliveira de Sousa

**Affiliations:** 1 Graduate Program in Pharmaceutical Innovation, Federal University of Amapá (UNIFAP), Macapá 68903-419, Brazil; fabio@unifap.br; 2 Department of Pharmacy (DFAR), Faculty of Pharmacy, Dentistry and Nursing (FFOE), Federal University of Ceará (UFC), Fortaleza 60430-372, Brazil; 3 Department of Surfactants and Nanobiotechnology, Instituto de Química Avanzada de Cataluña, Centro Superior de Investigaciones Científicas IQAC-CSIC, 08034 Barcelona, Spain; zakaria.hafidi@cid.csic.es; 4 Laboratory of Quality Control, Bromatology & Microbiology, School of Pharmacy, Department of Biological & Health Sciences, Federal University of Amapá (UNIFAP), Macapá 68903-419, Brazil

**Keywords:** arginine, surfactants, antimicrobial, antibiofilm, resistance, synergism

## Abstract

The rise in antimicrobial resistance and the resilience of microbial biofilms demand innovative strategies that combine, for instance, membrane-active agents with marketed drugs. Arginine-based surfactants are promising alternatives to conventional quaternary ammonium compounds, but comparative data on their antimicrobial, antibiofilm and modulatory activities remain limited. Five arginine-derived surfactants, the single-chain N*α*-lauroyl-L-arginine methyl ester (LAM) and ethyl ester (LAE), together with their double-chain homologues LANHC_3_, LANHC_5_ and LANHC_8_ were evaluated against Gram-positive and Gram-negative bacteria and four *Candida* spp. Minimum inhibitory (MIC) and lethal (MLC) concentrations were determined by broth microdilution method. Antibiofilm activity was assessed through minimum biofilm inhibitory (MBIC) and eradication (MBEC) concentrations. Checkerboard assays were used to evaluate the synergism between the surfactants and conventional therapeutic antibacterial and antifungal agents. LANHC_3_ and LANHC_8_ exhibited uniform antibacterial MICs of 19.53 µg/mL, while LAM and LANHC_5_ showed MICs of 19.53 µg/mL for most strains, with *Enterococcus faecalis* requiring 39.06 µg/mL. LANHC_3_ was the most potent surfactant over *Candida* spp. With MICs of 9.76 µg/mL for all species, and similarly to LAM, both were fungicidal at 39.06 µg/mL. LAM and LANHC_3_ also showed the lowest MBIC and MBEC values, inhibiting the *Candida* biofilm formation at 39.06 µg/mL and eradicating mature biofilms at 78.12 µg/mL, while the other surfactants required higher concentrations to disrupt the microbial biofilms. Synergic or additive interactions were found between the surfactants and selected β-lactam and macrolide antibiotics, as well as azole antifungals, with no antagonism observed. LAM and particularly LANHC_3_ combined broad-spectrum antimicrobial activity, relevant antibiofilm effects and the ability to potentiate the activity of conventional agents, supporting their choice as alternative or complementary antimicrobial adjuvants over resistant microorganisms and their biofilms.

## 1. Introduction

Traditional antimicrobial therapies are becoming progressively less effective against bacterial and fungal infections, primarily due to the rising prevalence of antimicrobial resistance triggered by Gram-positive bacteria (e.g., methicillin-resistant *Staphylococcus aureus*, vancomycin-resistant *Enterococcus* sp.), Gram-negative bacteria (e.g., *Pseudomonas aeruginosa, Acinetobacter baumannii*) and fungi (e.g., *Candida auris*) [1] combined with the irrational use of antimicrobial agents. Hence, the development of new antimicrobial alternatives is urgently necessary [1,2].

The spread of microbial resistance represents a serious risk to the humanity. Antimicrobial resistance (AMR) represents one of the most critical health challenges of the 21st century, associated with approximately 4.95 million deaths in 2019 and projected to reach up to 10 million deaths annually by 2050 if no effective measures are implemented [3,4]. The increasing inefficacy of conventional antibiotics is closely related to the upsurge of microbial biofilms, organized communities embedded in a self-produced extracellular polymeric matrix, which protect the microorganisms against host defenses and antimicrobial agents [5,6]. Biofilm-associated infections are notoriously difficult to eradicate, often requiring 100–1000× higher drug concentrations than their planktonic counterparts [7,8].

The development of new antimicrobial materials has therefore shifted toward biocompatible, biodegradable, and sustainable alternatives capable of preventing or disrupting biofilms without contributing to environmental toxicity or microbial resistance, aligned with the principles of green chemistry [9] and the development of sustainable surfactants [10]. Among these, amino acid-based surfactants have emerged as particularly promising molecules may be associated with their amphiphilic nature, tunable hydrophobic-hydrophilic balance and inherent biodegradability [11,12,13]. Derived from natural amino acids such as arginine, lysine, and histidine, these surfactants combine physicochemical versatility with membrane-targeting antimicrobial activity. For instance, the positively charged guanidinium group found in arginine surfactants allows strong electrostatic interactions with the negatively charged microbial membranes, leading to membrane permeabilization and cellular disruption [14,15].

**Figure 1:**
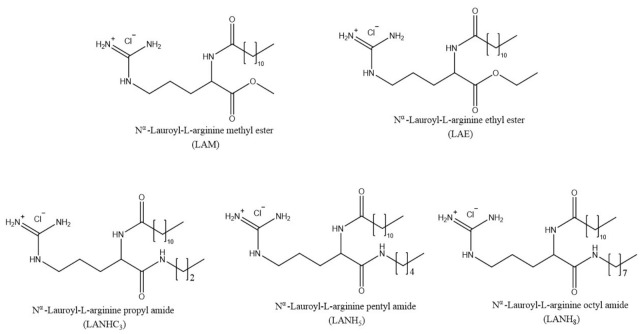
Chemical structures of the arginine-derived cationic surfactants LAM, LAE, LANHC_3_, LANHC_5_ and LANHC_8_.

Cationic surfactants derived from arginine, such as N*α*-lauroyl-L-arginine methyl ester (LAM), N*α*-lauroyl-L-arginine ethyl ester (LAE), N*α*-lauroyl-L-arginine propyl amide (LANHC_3_), N*α*-lauroyl-L-arginine pentyl amide (LANHC_5_) and N*α*-lauroyl-L-arginine octyl amide (LANHC_8_) (Figure 1), exhibit broad-spectrum antimicrobial activity against both Gram-positive and Gram-negative bacteria, as well as fungi including *Candida* spp. [13,16].

Upon the increment of the hydrophobic chain length or the introduction of a second short alkyl chain, the antimicrobial potency was found to increase, while their biodegradability and low cytotoxicity were maintained [13,16]. Despite their strong activity, bulk surfactants often face limitations related to instability, aggregation and interaction with biological components, which can reduce their efficacy in complex biological environments.

Recent studies have expanded the structural design through the synthesis of a new series of double-chain arginine-based surfactants (LANHC_3_, LANHC_5_ and LANHC_8_) (Figure 1), where a second alkyl chain (C_3_–C_10_) is linked to the lauroyl-arginine scaffold via amide bonding. These compounds show tunable hydrophobic–hydrophilic balance, lower critical micelle concentrations (0.2–5.4 mM), and superior surficial activity compared with the single-chain analogues [13]. In general, these double-chain surfactants demonstrated stronger bactericidal and antifungal effects, as well as pronounced antibiofilm activity, effectively eradicating mature *Staphylococcus* spp. and *Candida* spp. biofilms at very low concentrations [16]. In addition to their biological performance, these novel surfactants showed up to 63% biodegradability and a ten-fold lower aquatic toxicity compared with traditional quaternary ammonium compounds. For instance, over *Daphnia magna* they reached IC_50_ values above 4 mg/L and negligible toxicity toward marine microalgae [13].

Regarding the safety profile of arginine-derived surfactants for potential human use, previous studies have demonstrated low cytotoxicity against mammalian cell lines, including fibroblasts and keratinocytes [17,18]. In general, these cationic surfactants exhibit moderate to low hemolytic activity with HC_50_ values > 32 µM [17]. In our previously published paper, the therapeutic index (TI) of that chemical family was also calculated. Larger TI values indicate better selectivity against bacterial membranes [19,20,21]. Overall, these compounds caused lysis only at concentrations well above their MIC values against both bacteria and fungi [16]. Compounds LAM, LAE and LANHCn (n = 3–6) exhibited a similar safety window, with TI values between 3 and 6. In these homologs, the antimicrobial efficiency increased by the elongation of the second alkyl chain, but the hemolytic character also increased at a comparable rate [16]. Compared to the other compounds, LANHC_8_ presented a notably lower hemolytic nature and its high HC_50_ values resulted in higher TI values, indicating an improvement in its selectivity. The TI values observed for these surfactants can be ascribed to an optimum charge/hydrophobicity balance, which can contribute to their selectivity towards bacterial membranes [13,22]. Additionally, these compounds are readily biodegradable via amidase cleavage of the amide bond between the arginine head and the fatty acid chain [11]. Although further in vivo studies are required, the available in vitro data suggest a favorable toxicological profile for topical or controlled-release applications.

Their ecological safety and ability to inhibit metal corrosion (up to 80% efficiency on carbon steel at 0.5 mM) further highlight their projection and environmental compatibility, reinforcing their potential as the next generation of sustainable cationic agents [13]. These findings consolidate the position of arginine-based surfactants as biocompatible, biodegradable and multifunctional antimicrobial candidates, capable of addressing not only clinically relevant infections but also environmental and industrial safety concerns. While corrosion inhibition and low ecotoxicity are valuable for industrial and environmental applications, they do not directly contribute to the antimicrobial mechanism, which relies primarily on membrane disruption by cationic monomers.

In addition to the intrinsic activity of new molecules, combined therapies and the use of adjuvants that potentiate conventional antibiotics have gained attention as a smart strategy to enhance efficacy, restore the activity of existing drugs and reduce their doses. Membrane-active surfactants are particularly attractive in this context because they can facilitate drug penetration and disturb the biofilms’ architecture, potentially improving the antimicrobial performance and reducing microbial resistance mechanisms.

In this context, the present study aimed to compare the antimicrobial and antibiofilm activities of five biodegradable arginine-derived surfactants (LAM, LAE, LANHC_3_, LANHC_5_ and LANHC_8_) against clinically relevant Gram-positive and Gram-negative bacteria and *Candida* spp. together with their ability to modulate the activity of selected antibacterial and antifungal agents in planktonic cultures and preformed biofilms.

## 2. Results and Discussion

### 2.1. Antimicrobial Activity

**Figure 2:**
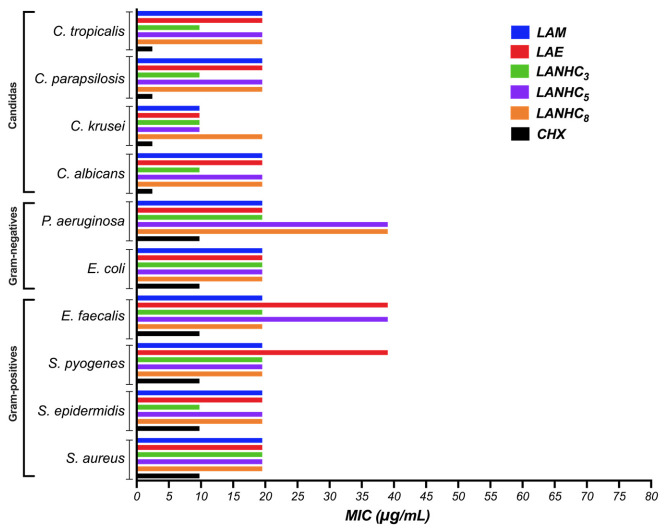
Minimum inhibitory concentration (MIC, µg/mL) of the arginine-based surfactants LAM, LAE, LANHC_3_, LANHC_5_, LANHC_8_ and chlorhexidine gluconate (CHX) against Gram-negative, Gram-positive and *Candida* spp. strains. Data are expressed as mean of three independent experiments (n = 3).

**Figure 3:**
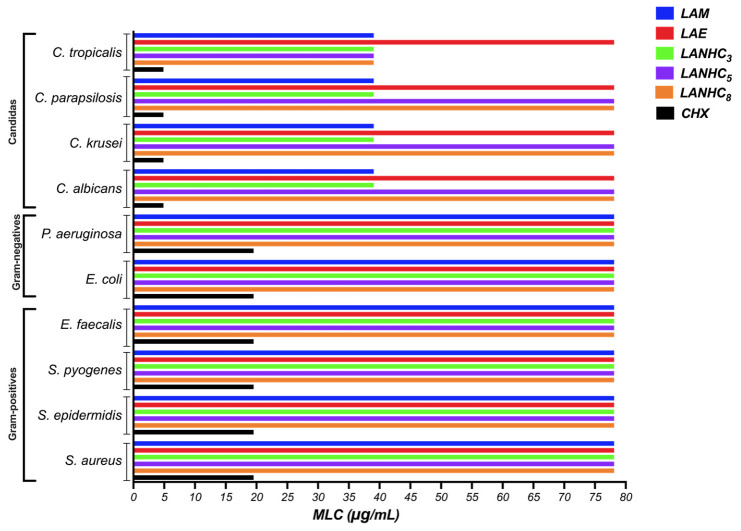
Minimum lethal concentration (MLC, µg/mL) of the arginine-based surfactants LAM, LAE, LANHC_3_, LANHC_5_, LANHC_8_ and chlorhexidine gluconate (CHX) against Gram-negative, Gram-positive and *Candida* spp. strains. Data are expressed as mean of three independent experiments (n = 3).

The MIC and MLC values for the surfactants are shown in Figure 2 and Figure 3. The MIC values for CHX (positive control) against both ATCC Gram-positive and Gram-negative bacterial strains were 9.76 μg/mL. Moreover, the *Candida* spp. were found more susceptible to CHX, showing MIC values of 2.44 μg/mL, in agreement with the literature [23].

The surfactants LANHC_3_ and LANHC_8_ showed similar antimicrobial activity against all Gram-positive and Gram-negative strains (MIC = 19.53 μg/mL). The similarity was also observed for LAM and LANHC_5_, except for *E. faecalis*, whose MIC increased to 39.06 μg/mL. This result follows the trend observed in recent studies [16], where the antimicrobial activity of N*α*-lauroyl-L-arginine N-alkylamide homologues demonstrated nonlinear dependence on the hydrophobic chain length, with an optimal activity within the intermediate chain lengths.

The result observed against *E. faecalis* can be explained by the intrinsic resistance of this strain compared to other Gram-positive bacteria [24]. In the study of Pereira et al., (2024) [25] demonstrated that *E. faecalis*, without previous exposure to antimicrobials, had significantly higher MIC (2–8×) than other Gram-positive strains, confirming its intrinsically resistant characteristics.

Also, *E. faecalis* naturally has higher resistance to surfactants due to the presence of uniquely composed peptidoglycan (D-Asp/D-Asn bonds and 3→3 cross-linking); the abundance of distinctive lipoteichoic acids (saturated diacylglycerol anchors); specific surface proteins such as *Enterococcus* collagen (Ace) and aggregation substance (AS); high concentration of cardiolipin (25%) and asymmetric distribution of phospholipids in the membrane, in addition to the secretion of constitutive proteases such as Gelatinase (GelE) and Serine Protease (SprE) that may degrade peptide surfactants [24,25].

Among the Gram-negative strains, *P. aeruginosa* lead to higher MIC values (39.06 μg/mL) when treated with LAE, LANHC_5_ and LANHC_8_ than *E. coli* (MIC = 19.53 μg/mL). This result may be partly explained mainly by structural differences found in the outer membrane of *P. aeruginosa*. This naturally occurring aspect shows a permeability 10–100 times lower than that of *E. coli*, due to the distinguished lipidic composition and the more compact molecular arrangement [26,27]. *E. coli* porins (mainly *OmpF* and *OmpC*) form relatively larger and more abundant channels, facilitating the entry of drugs, while *P. aeruginosa* constitutively expresses more selective porins and in smaller extents [27,28,29,30].

Moreover, *P. aeruginosa* has more efficient constitutive efflux pump systems, such as *MexAB-OprM*, which actively expel drugs before they reach intracellular toxic concentrations [28]. Chromosomal β-lactamases, although present in both species, are more diverse and expressed in higher levels in *P. aeruginosa* [27,31]. Despite the biological difference in these two Gram-negative bacteria, the results for LANHC_3_ and LAM were found similar (MIC = 19.53 μg/mL), suggesting that these surfactants were able to overcome the challenges inherent to *P. aeruginosa*.

The best activity against the Candida strains was observed for LANHC_3_ (MIC = 9.76 μg/mL). LAM, LANHC_5_ and LANHC_8_ showed similar MIC values (9.76 μg/mL) against *Candida krusei*, while the MIC increased to 19.53 μg/mL over the other stains tested. Therefore, despite its intrinsic resistance to Fluconazole, *C. krusei* was the most sensitive strain among the yeasts [32]. This phenomenon was also observed for miconazole, despite the presence of two chlorinated aromatic rings, as demonstrated by Campos Péret et al. (2023) [33]. In that study, *C. krusei* showed a greater susceptibility to miconazole with a MIC of 18.7 μM, substantially lower than the value observed for *C. albicans* (150.2 μM) and *C. auris* (74.9 μM) [34].

The cell wall of *C. krusei* has a higher exposure of β-1,3-glucans on the surface, making it more susceptible to some antifungals [33]. In addition, its cell membrane has a differentiated distribution of ergosterol, while its greater permeability facilitates the penetration of active ingredients [35,36]. This set of factors may contribute to the lower MIC values obtained. Among the LANHC series, LANHC_3_ (C3 spacer) showed the lowest MIC against *C. krusei* (9.76 µg/mL), while LANHC_5_ and LANHC_8_ required higher concentrations (19.53 µg/mL). This difference may be attributed to the higher molecular mobility and greater monomer availability of LANHC_3_ due to its higher CMC, whereas the longer and more hydrophobic spacers of LANHC_5_ and LANHC_8_ favor micellization, reducing the effective monomer concentration at the fungal cell wall.

LAE showed slightly higher MIC values (39.06 μg/mL), over all Gram-positive and Gram-negative bacteria and most yeasts. The lowest value observed for LAE was solely against *C. krusei*, with an MIC of 19.53 μg/mL. However, despite this difference, the MIC values found are consistent with previous studies [37].

Regarding the concentration required to eradicate the microorganisms (MLC) (Figure 3), the MLC results obtained were similar among the five surfactants (MLC = 78.12 μg/mL) against all Gram-positive and Gram-negative bacteria. Against the fungal strains, the best performance was reached by LAM and LANHC_3_, with MLC of 39.06 μg/mL against the four strains tested. While LANHC_5_ and LANHC_8_ reached this value only against *C. tropicalis*. The MLC over the other strains was 78.12 μg/mL, similarly to LAE. The positive control CHX presented a MLC of 19.53 μg/mL and 4.88 μg/mL for bacteria and fungi, respectively.

In summary, LAM and LANHC_3_ presented the highest antimicrobial potency, followed by LANHC_5_ and LANHC_8_, with slightly lower results. This effect can be explained by either the intrinsic properties of the microorganisms evaluated or by the molecular characteristics of each surfactant, for instance the aggregation behavior, represented by different CMC values [38,39,40]. The results found in this manuscript are slightly different from those found in our previous study [16], where LANHC_5_ and LANHC_6_ were found the most potent molecules within this group. Those results indicated that the antimicrobial activity increased as the alkyl chain length increased up to the C6 homolog. Therefore, the small differences among LAM, LAE C3, C5 and C6 homologues can result in similar or alternating activities, according to the tested conditions. However, for the C8 and C10 homologs, it was found to decrease [13]. It should be noted that both the cytotoxicity and the antimicrobial activity of membrane-disrupting antimicrobials such as cationic surfactants are driven by the same structural parameters; the hydrophobic alkyl chain and the cationic charge [16]. Usually, for the same cationic charge, the cytotoxicity of cationic surfactants increases as their hydrophobic content increases [13,15,16].

LAM, with its C12 chain and methyl ester group, may maintain a high concentration of active monomers below or near its CMC (2.73 µg/mL), thereby favoring free interaction with the bacterial membrane [41,42].

The structural conformation of LANHC_3_, with a shorter spacer chain (3 carbons) and 2 nonpolar chains similar to LAM, linked to the amino acid arginine, maintains its molecular mobility, which results in a high solubility and a very similar CMC (2.34 µg/mL), ensuring the availability of active monomers at moderate concentrations [39,43]. Despite its similar molecular structure to LAM, it was found to be more susceptible to interactions with proteins and peptides in the environment, which may explain its slightly lower performance than its pairs [40,44].

The surfactants LANHC_5_ and LANHC_8_, with structural similarity to LANHC_3_, with chains of 5 and 8 C, respectively, are found more hydrophobic and, as a consequence, present lower molecular mobility, which results in lower CMC values (1.02 and 0.202 µg/mL, respectively) [45]. This characteristic results in a lower availability of active monomers in solution and consequently in an intermediate antimicrobial activity. While LANHC_5_ still maintain a balance between solubility and membrane interaction, LANHC_8_, with its longer chain, tends to form micelles more easily, reducing the availability of active monomers [20].

The distinguished performance of the N*α*-lauroyl-L-arginine N-alkylamide homologues in our study corroborates the concept of “window of activity” described by Hafidi et al. (2025), where the antimicrobial efficacy increases with the length of the chain to an optimum, gradually decreasing for longer chains [16]. Unlike that study, the optimum results in our study were found for LANHC_3_, possibly due to factors related to the molecular mobility, controlled aggregation patterns and also related to the strains and methodology tested. The MLC/MIC ratio for all compounds remained in the range of 1–2, reinforcing their bactericidal and fungicidal nature, in agreement with the findings of Hafidi et al. [16] for the complete LANHCx series.

### 2.2. Inhibitory and Eradication Biofilm Activity

**Figure 4:**
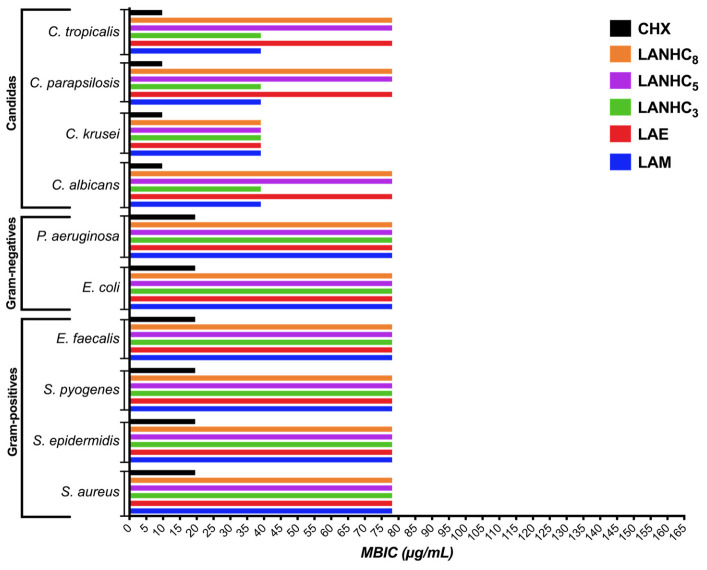
Minimum biofilm inhibitory concentration (MBIC, µg/mL) of LAM, LAE, LANHC_3_, LANHC_5_, and LANHC_8_ against Gram-negative, Gram-positive bacteria and *Candida* spp. biofilms. Data are expressed as mean of three independent experiments (n = 3).

**Figure 5:**
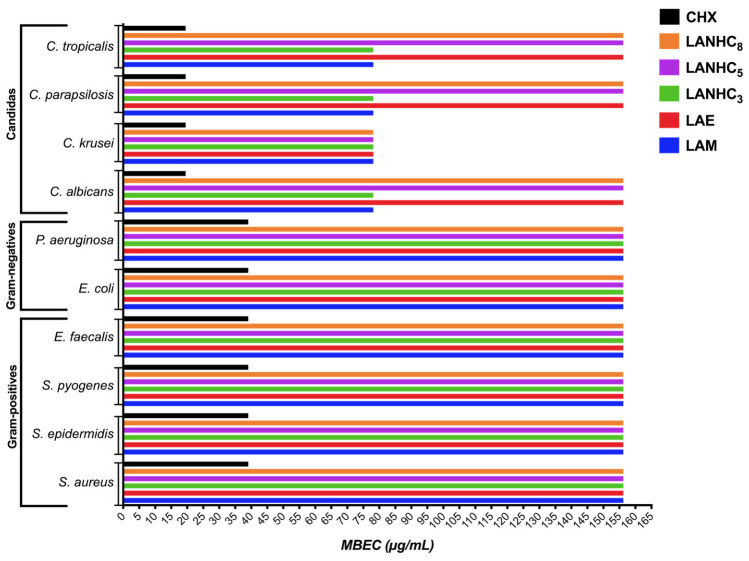
Minimum biofilm eradication concentration (MBEC, µg/mL) of LAM, LAE, LANHC_3_, LANHC_5_, and LANHC_8_ against Gram-negative, Gram-positive bacteria and *Candida* spp. biofilms. Data are expressed as mean of three independent experiments (n = 3).

The activity of the surfactants against bacteria and yeasts biofilms is expressed in terms of MBIC and MBEC (Figure 4 and Figure 5). LAM and LANHC_3_ were found slightly more active against the bacteria and *Candida* spp. biofilms.

This aspect can be explained by the structural differences and composition of these structures, as well as the mechanism of action of these compounds. While LAM and LANHC_3_ demonstrated high activity against isolated bacterial and fungal cells, as previously discussed, their performance over biofilms is influenced by the presence of extracellular matrix and its complexity together with the cell membrane sensitivity [46,47,48]. Differences in the matrix composition between *Candida* spp. and bacterial biofilms may influence surfactant penetration. The Candida matrix, which can be less negatively charged, is composed mainly of polysaccharides such as β-glucans, a feature that may facilitate the penetration of the surfactants in certain cases [49]. In addition, the ergosterol-rich fungal cell membrane may be more sensitive to the action of cationic surfactants, allowing LAM and LANHC_3_ to interact efficiently and cause their destabilization [49].

In contrast, bacterial biofilms have a denser and complex extracellular matrix, rich in polysaccharides, proteins, and extracellular DNA, which acts as a physical and chemical barrier, making it more difficult for the drugs to penetrate [46,50]. Additionally, the bacterial cell membrane, especially in Gram-negative cells, is found to be more resistant due to the presence of an additional outer membrane composed of lipopolysaccharides (LPSs) [26]. These factors reduce the availability of active LAM and LANHC_3_ monomers within the bacterial biofilms, even at concentrations below CMC. In contrast, the *Candida* spp. biofilms, with lower matrix complexity and higher cell membrane sensitivity confirmed their superior antimicrobial activity [48,51].

A recent study reported the ability of N*α*-lauroyl-L-arginine N-alkylamide homologues to eradicate mature biofilms of *C. albicans* and *C. tropicalis* at concentrations of 32-64 μg/mL [16]. Those authors attributed this effect to the dense cationic load and the presence of guanidine and amide groups, which provided a broad spectrum of intermolecular interactions, facilitating the surfactants diffusion through the polymeric matrix of the biofilm [16].

The molecular structure of the surfactants, their CMC and the characteristics of the biofilms may explain why LAM and LANHC_3_ were found more effective over *Candida* spp. than against bacteria. In contrast, while LANHC_5_ and LANHC_8_, with larger spacer chains, have a moderate performance, and LAE, despite its good results in the literature, may have faced a reduction on their efficacy over bacterial biofilms due to the formation of micelles and their interactions with the extracellular matrix [52]. The findings against biofilms corroborate the results obtained for the microorganisms in the planktonic form, as previously described.

### 2.3. Combined Effect of the Surfactants with Clinical Antimicrobials

**Figure 6:**
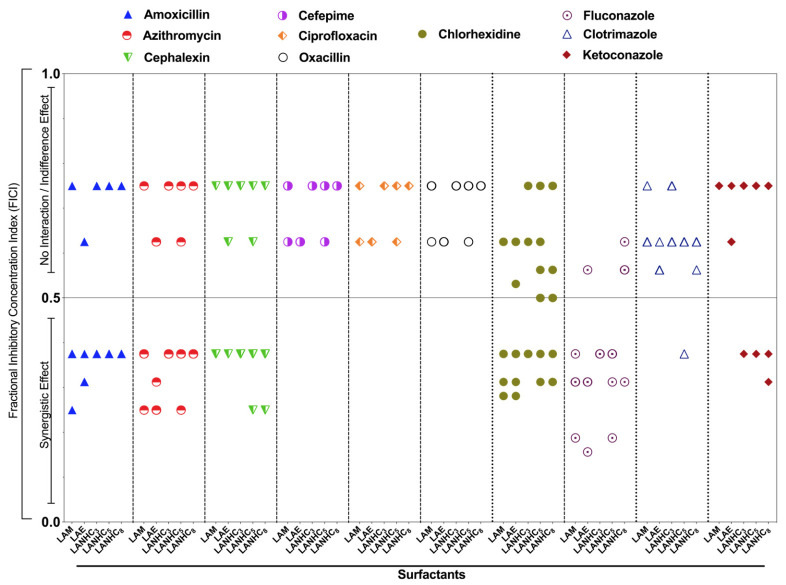
Graphical summary of synergistic interactions (FICI ≤ 0.5) between arginine-derived surfactants and clinically relevant antimicrobial agents. Amoxicillin, azithromycin, cephalexin, chlorhexidine, ketoconazole, clotrimazole, and fluconazole showed synergy with one or more surfactants, while no synergy was observed with cefepime, oxacillin, or ciprofloxacin. The complete dataset with exact FICI values for each microbial strain is available in Supplementary Table S1.

Regarding the modulatory activity of the surfactants combined with clinical used antimicrobials, it was possible to observe that the arginine-derived surfactants were able to synergistically modulate the action of several agents against Gram-positive and Gram-negative bacteria and fungi, demonstrated by the significative reduction in the MIC values. The synergism was evident between the surfactants and antimicrobial drugs such as amoxicillin, azithromycin and cephalexin (Figure 6, Table S1). Contrariwise, no synergism was found in the combinations with oxacillin, cefepime and ciprofloxacin. Moreover, the synergic effect was evidenced with the antifungals: ketoconazole, clotrimazole, and fluconazole (Figure 6, Table S1).

The synergism between the cationic surfactants (LAM, LAE, LANHC_3_, LANHC_5_, LANHC_8_) and the antimicrobials (azoles, chlorhexidine, cephalexin, azithromycin, and amoxicillin) may have occurred because the first would increase the permeability of the cellular membrane, facilitating the access and activity of these agents [53,54].

In contrast, for combinations involving cefepime (zwitterionic) or oxacillin (sodium salt), no synergistic effects were observed. One possible explanation is chemical incompatibility between the cationic surfactants and these antibiotics. Specifically, electrostatic interactions between the positive charges of the surfactants and the carboxylate group (-COO^−^) of cefepime, or the carboxylate (-COO^−^Na^+^) of oxacillin, could potentially lead to the formation of insoluble complexes or precipitates, thereby reducing the bioavailability of both compounds. However, further physicochemical studies (e.g., turbidity assays, precipitation tests) are needed to confirm this hypothesis [55,56,57]. In these cases, the interaction promotes the loss of activity, which may explain the absence of synergism [57,58]. Another independent explanation, particularly for ciprofloxacin, is based on its high intrinsic efficacy and intracellular mechanism of action, which would limit any additional contribution from surfactants [59,60,61].

The combinations containing chlorhexidine demonstrated broad synergistic activity against both bacterial and fungal strains, including Gram-positive bacteria, *E. coli*, *P. aeruginosa*, and several Candida species. These findings highlight the potential of arginine-derived surfactants to enhance the antimicrobial performance of chlorhexidine across a wide spectrum of clinically relevant microorganisms. Furthermore, the observed synergism suggests that lower concentrations of chlorhexidine could potentially be used in antimicrobial formulations while maintaining efficacy. This may represent an important advantage in reducing adverse effects commonly associated with chlorhexidine, such as mucosal irritation, tooth staining, taste alteration, and cytotoxicity related to prolonged or high-concentration exposure [62].

The combined use of two or more antimicrobial agents, profiting from their synergism represents a great potential and at the same time, an opportunity to face the growing phenomenon of microbial resistance. The associations can hinder the emergence of defense mechanisms, which would become much more complex to neutralize [63,64]. In addition, the synergy allows the same efficacy to be achieved with reduced doses of each individual compound, reducing the dose-related toxicity associated with the use of single agents [65]. Finally, the combination can increase the range of antimicrobial activity, making the treatment effective against a wider range of microorganisms, including those resistant to conventional treatments [63] (Figure 6, Table S1). Relevant synergistic interactions (FICI ≤ 0.5) between arginine-based surfactants and clinically relevant antimicrobial drugs obtained upon the cross-board check assay.

## 3. Materials and Methods

### 3.1. Materials

A total of 10 American Type Culture Collection (ATCC) strains were used for the antimicrobial and antibiofilm assays. All microorganisms were obtained from the Oswaldo Cruz Foundation (FIOCRUZ, Rio de Janeiro, Brazil). Those included both Gram-positive and Gram-negative bacteria, as well as yeast species commonly associated with biofilm-related infections. The following strains were tested: *Staphylococcus aureus* ATCC 25923*, Staphylococcus epidermidis* ATCC 35984, *Streptococcus pyogenes* ATCC 19615, *Enterococcus faecalis* ATCC 14506, *Escherichia coli* ATCC 25922, *Pseudomonas aeruginosa* ATCC 27853, *Candida albicans* ATCC 10231, *Candida krusei* ATCC 6258, *Candida parapsilosis* ATCC 22019, and *Candida tropicalis* ATCC 20336. The culture media Brain–Heart Infusion Broth (BHI), Brain–Heart Agar (BHA), Casein–Soy Broth (CSB) and Agar (CSA), Sabouraud Broth (SB) and Agar (SA), Mueller–Hinton Broth (MHB) and Agar (MHA), Plate Count Agar (PCA), and Phosphate-Buffered Saline (PBS) were obtained from Merck^®^ (Darmstadt, Germany). Chlorhexidine gluconate (CHX), Amoxicillin, Azithromycin, Cephalexin, Cefepime, Oxacillin, Ciprofloxacin, Chlorhexidine digluconate, Ketoconazole, Fluconazole and Clotrimazole were purchased from Sigma-Aldrich^®^ (St. Louis, MO, USA).

All surfactants were synthesized at the Institute of Advanced Chemistry of Catalonia (IQAC-CSIC, Barcelona, Spain), according to our previous published paper [16]. They were prepared from the corresponding unprotected amino acid through a simple and economical two-step sequence: (a) preparation of lauroyl arginine methyl ester (LAM) or ethyl ester (LAE) from arginine methyl/ethyl ester and lauroyl chloride, and (b) reaction of LAM with propylamine, pentylamine or octylamine, without the use of any solvent, to obtain the compounds LANHC_3_, LANHC_5_ and LANHC_8_ [16].

This synthetic strategy offers an efficient and sustainable approach to the preparation of antimicrobial compounds. The process is based on renewable starting materials and is characterized by high atom economy, reduced waste generation, and minimal consumption of organic solvents, thereby aligning with the principles of green chemistry. Finally, the purity of the compounds was evaluated by HPLC, mass spectrometry, and NMR spectroscopy. The purity of each compound was confirmed by HPLC analysis, and all compounds were used at ≥95% purity.

The following arginine-derived surfactants (Figure 1) were used in this study: Nα-lauroyl-L-arginine methyl ester (LAM), Nα-lauroyl-L-arginine ethyl ester (LAE), Nα-lauroyl-L-arginine propyl amide (LANHC_3_), Nα-lauroyl-L-arginine pentyl amide (LANHC_5_), and Nα-lauroyl-L-arginine octyl amide (LANHC_8_).

### 3.2. Preparation and Standardization of the Microbial Inoculum

Each microbial strain was sub-cultured from a stock culture into 10 mL of MHB for bacteria or SB for fungi and incubated overnight at 37 °C. After that, a small aliquot was transferred into sterile saline solution (0.85% *w*/*v* NaCl) and adjusted spectrophotometrically to an absorbance of 0.8–1.0 at 620 nm, corresponding to 0.5 McFarland standard, equivalent to approximately 1.5 × 10^8^ CFU/mL for bacteria and 1.5 × 10^6^ CFU/mL for fungi [66,67].

### 3.3. Determination of Minimum Inhibitory (MIC) and Minimum Lethal (MLC) Concentrations

MIC values, defined as the lowest concentration capable of inhibiting visible microbial growth, were determined by broth microdilution in sterile 96-well microplates (300 µL capacity), following CLSI M07 for aerobic bacteria and CLSI M27 for yeasts, with adaptations for the tested surfactants [68,69]. Each well contained 80 µL of MHB for bacteria or SB for fungi, 100 µL of the test samples (surfactants) or the standard antimicrobial (CHX), serially diluted in twelve concentrations ranging from 0.305 to 625 µg/mL, and 20 µL of the microbial inoculum (adjusted to 10^6^ CFU/mL). Negative and positive controls consisted of culture medium, diluent, and inoculum without treatment. Plates were incubated at 37 °C for 24 h. The MIC was defined as the lowest concentration with no visible turbidity. To confirm the MIC values, a colorimetric resazurin assay was performed to eliminate false positives caused by the intrinsic turbidity of the samples [17]. After incubation, 20 µL of 0.015% (*w*/*v*) resazurin solution were added to each well and incubated for 30 min at 37 °C. Wells that remained blue indicated absence of metabolic activity, while the change to pink/purple denoted viable cells. After the MIC determination, MLC values were established as the lowest concentration capable of eliminating 99.9% of viable cells [17,68]. Ten microliters from each MIC well and the two subsequent concentrations above were spread onto appropriate agar plates (MHA or SA). Plates were incubated at 37 °C for 24h, and the MLC was recorded as the lowest concentration with no visible colonies. All assays were performed in triplicate.

### 3.4. Determination of Minimum Biofilm Inhibitory Concentration (MBIC)

The minimum concentration inhibiting biofilm formation (MBIC) was determined by the crystal violet staining method, adapted from previous reports [68,69].

The ten strains selected (listed in Section 3.1) were grown in MHB or SB supplemented with 1% glucose and incubated at 37 °C for 24 h. Afterwards, 180 µL of microbial suspension (10^6^ CFU/mL) and 20 µL of the surfactants or chlorhexidine solutions (4.88–625 µg/mL) were added to the respective wells of a 96-well microplate. After 24 h at 37 °C, non-adherent cells were removed by gentle washing with sterile saline solution (0.85% *w*/*v* NaCl).

The adherent cells were fixed with 99% methanol for 15 min and air-dried. After that, the biofilms were stained with 50 µL of 0.1% (*w*/*v*) crystal violet for 25 min, rinsed with water, and the bound dye was solubilized with 150 µL of 96% ethanol containing 10% acetic acid. The absorbance was measured at 570 nm using an Agilent BioTek 800 TS microplate reader (Agilent Technologies, Santa Clara, CA, USA). MBIC was defined as the lowest concentration producing a statistically significant reduction (*p* < 0.05) in the mean absorbance versus untreated controls.

### 3.5. Determination of Minimum Biofilm Eradication Concentration (MBEC)

The MBEC is defined as the minimum concentration required to eradicate pre-formed biofilms. It was determined using a modified broth microdilution method [17,69]. The 10 microbial strains referred to in Section 3.1 were cultured in MHB or SB supplemented with 1% glucose and incubated at 37 °C for 24 h. Aliquots of 100 µL of each culture (10^6^ CFU/mL) were transferred to sterile 96-well plates and incubated for 48 h to promote biofilm formation. The wells were washed twice with 200 µL of ultrapure water to remove planktonic cells. After that, 100 µL of each treatment (surfactants or chlorhexidine solutions) in different concentrations (4.88–625 µg/mL) and 100 µL of fresh glucose-supplemented medium were added to each well, followed by incubation at 37 °C for 24 h. After that, the wells were washed with 200 µL of ultrapure water. The remaining adhered biofilm cells were detached by gently scraping using a sterile micropipette tip, resuspended in 0.85% sterile saline solution, and homogenized in a vortex for 30 s. Serial dilutions were performed, and 5 µL aliquots (microdropper method) [69] were plated onto the suitable agar (MHA, PCA, or SA). The plates were incubated at 37 °C and evaluated after 24 or 48h for bacterial and fungal strains, respectively. The MBEC was defined as the lowest concentration that eradicated pre-formed biofilms, corresponding to a ≥99% reduction in viable colonies compared with untreated controls.

### 3.6. Combined Activity of Surfactants with Conventional Antimicrobials

The modulatory effect of surfactants on the activity of clinically relevant antimicrobials (amoxicillin, azithromycin, cephalexin, cefepime, oxacillin, ciprofloxacin, chlorhexidine, ketoconazole, fluconazole, and clotrimazole) was assessed using the checkerboard microdilution method [70,71,72]. Aliquots of 20 μL of microbial cultures (10^6^ CFU/mL), 80 μL of MHB or SB, 50 μL of surfactant solutions, and 50 μL of each antimicrobial agent were added to 96-well microplates. The final concentrations of the surfactants and antimicrobial agents corresponded to 1/2, 1/4, 1/8, and 1/16 of the previously determined MIC values. After incubation at 37 °C for 24 h, microbial growth was evaluated visually by turbidity and confirmed by resazurin assay when necessary.

To evaluate the effect of the combinations, the fractional inhibitory concentration index (FICI) was calculated according to Equation (1) [71]:

(1) $$FICI=FIC_{Surfactant}+FIC_{ATB}=\frac{\left[Surfactant\right]}{MIC_{Surfactant}}+\frac{\left[ATB\right]}{MIC_{ATB}}$$

where FIC (Fractional Inhibitory Concentration) for each agent is defined as the ratio between the concentration of the agent used in combination and its minimum inhibitory concentration (MIC) when tested alone. Thus, [Surfactant] and [ATB] represent the concentrations of the surfactant, and the antimicrobial used in combination, respectively, while MIC Surfactant and MIC ATB represent their individual MIC values.

The FICI was interpreted according to commonly used checkerboard criteria: synergy was defined as FICI ≤ 0.5, no interaction/indifference as 0.5 < FICI ≤ 4.0, and antagonism as FICI > 4.0 [73,74]. The assays were performed in triplicate for each microorganism.

### 3.7. Statistical Analysis

All experiments were performed in triplicate in at least three independent assays. Data are expressed as mean out of three repetitions. Differences between the treatments and the positive control were analyzed by one-way ANOVA followed by Tukey’s post hoc test, using *p* < 0.05 as the criterion for statistical significance. Statistical analyses were performed using the software GraphPad Prism version 9.0 (San Diego, CA, USA).

## 4. Conclusions

In conclusion, this study demonstrates three key findings: (1) among the five surfactants evaluated, LAM and LANHC_3_ exhibited the strongest antimicrobial activity, with MIC values as low as 9.76 μg/mL against *Candida* spp. and 19.53 μg/mL against most bacteria; (2) both LAM and LANHC_3_ effectively eradicated pre-formed *Candida* biofilms at 78.12 μg/mL, supporting their potential for biofilm-related infections; (3) synergistic interactions were observed between the surfactants and amoxicillin, azithromycin, cephalexin, and azole antifungals (FICI ≤ 0.5), with no antagonism detected. These results position LAM and LANHC_3_ as promising candidates for further development as antimicrobial adjuvants, particularly in biofilm control and combination therapy.

## Data Availability

The original contributions presented in this study are included in the article/Supplementary Materials. Further inquiries can be directed to the corresponding authors.

## Supplementary Materials

The following supporting information can be downloaded at https://www.mdpi.com/article/10.3390/ijms27135936/s1.

## Abbreviations

AMR	Antimicrobial Resistance
ATCC	American Type Culture Collection
CFU	Colony Forming Units
CHX	Chlorhexidine digluconate
CLSI	Clinical and Laboratory Standards Institute
EPS	Extracellular Polymeric Substance
FDA	U.S. Food and Drug Administration
FIOCRUZ	Oswaldo Cruz Foundation
GRAS	Generally Recognized as Safe
LAM	N-lauroyl-L-arginine methyl ester
LAE	N-lauroyl-L-arginine ethyl ester
LANHC_3_	N-lauroyl-L-arginine N-propylamide
LANHC_5_	N-lauroyl-L-arginine N-pentamide
LANHC_8_	N-lauroyl-L-arginine N-octylamide
MIC	Minimum Inhibitory Concentration
MLC	Minimum Lethal Concentration
MBIC	Minimum Biofilm Inhibitory Concentration
MBEC	Minimum Biofilm Eradication Concentration
NaOH	Sodium Hydroxide
PBS	Phosphate-Buffered Saline
SA	Sabouraud Agar
SB	Sabouraud Broth
D-Asp/D-Asn	D-aspartate/D-asparagine
Ace	Adhesin to collagen of Enterococcus
GelE	Gelatinase (metallo-protease) of *E. faecalis*
SprE	Serine protease of *E. faecalis*
OmpC	Outer membrane porin C of *E. coli*
OmpF	Outer membrane porin F of *E. coli*
MexAB-OprM	A tripartite multidrug efflux pump of *P. aeruginosa* (MexA: membrane fusion protein; MexB: RND transporter; OprM: outer-membrane channel).
