# Supplementary material for: Single- and Double-Chain Arginine-Derived Surfactants: Antimicrobial, Antibiofilm and Synergistic Activities
Source: Int J Mol Sci. 2026 Jul 1;27(13):5936. doi: 10.3390/ijms27135936 (PMC13362268; doi:10.3390/ijms27135936)
Supplement: Supplementary file 1 [file ijms-27-05936-s001.zip › ijms-4353348-supplementary.pdf]

Table S1. Detailed FICI values for all combinations of arginine-derived surfactants and clinically relevant antimicrobial agents against each microbial strain. Synergy (FICI ≤ 0.5) is indicated in bold. No antagonistic interactions (FICI ≥ 1.0) were observed. See Materials and Methods (Section 3.6) for FICI calculation and interpretation.

Surfactants	Antimicrobial agents (FICI) [1]						
	**Amoxicillin**	**Azithromycin**	**Cephalexin**		**Ketoconazole**	**Clotrimazole**	**Fluconazole**
**LAM**	*S. aureus* (0.37); *S. epidermidis* (0.37); *S. pyogenes* (0.25); *E. faecalis* (0.37); *E. coli* (0.37)	*S. aureus* (0.37); *S. epidermidis* (0.31); *S. pyogenes* (0.25); *E. faecalis* (0.25); *E. coli* (0.25)	*S. aureus* (0.37); *S. epidermidis* (0.37); *S. pyogenes* (0.37); *E. faecalis* (0.37); *E. coli* (0.37)		–	–	*C. albicans* (0.31); *C. krusei* (0.37); *C. parapsilosis* (0.31); *C. tropicalis* (0.18)
**LAE**	*S. aureus* (0.31); *S. epidermidis* (0.37); *E. faecalis* (0.37); *E. coli* (0.31)	*S. epidermidis* (0.31); *S. pyogenes* (0.25); *E. faecalis* (0.25); *E. coli* (0.25)	*E. faecalis* (0.37)		–	–	*C. albicans* (0.31); *C. krusei* (0.31); *C. tropicalis* (0.15)
**LANHC$_3$**	*S. aureus* (0.37); *S. epidermidis* (0.37); *E. faecalis* (0.37); *E. coli* (0.37)	*S. aureus* (0.37); *S. epidermidis* (0.37); *E. faecalis* (0.25); *E. coli* (0.25)	*S. aureus* (0.37); *S. epidermidis* (0.37); *E. faecalis* (0.37); *E. coli* (0.37)		*C. krusei* (0.37)	–	*C. albicans* (0.37); *C. krusei* (0.37); *C. parapsilosis* (0.37); *C. tropicalis* (0.37)
**LANHC$_5$**	*S. aureus* (0.37); *S. epidermidis* (0.37); *S. pyogenes* (0.37); *E. faecalis* (0.37); *E. coli* (0.37)	*S. epidermidis* (0.37); *S. pyogenes* (0.37); *E. faecalis* (0.25); *E. coli* (0.37)	*S. aureus* (0.37); *S. epidermidis* (0.25); *S. pyogenes* (0.37); *E. faecalis* (0.37); *E. coli* (0.37)		*C. krusei* (0.37)	*C. krusei* (0.37)	*C. albicans* (0.37); *C. krusei* (0.37); *C. parapsilosis* (0.31); *C. tropicalis* (0.18)
**LANHC$_8$**	*S. aureus* (0.37); *S. epidermidis* (0.37); *S. pyogenes* (0.37); *E. faecalis* (0.37); *E. coli* (0.37)	*S. aureus* (0.37); *S. epidermidis* (0.37); *S. pyogenes* (0.37); *E. faecalis* (0.37); *E. coli* (0.37)	*S. aureus* (0.25); *S. epidermidis* (0.25); *S. pyogenes* (0.37); *E. faecalis* (0.37); *E. coli* (0.37)		*C. albicans* (0.37); *C. krusei* (0.31)	–	*C. krusei* (0.31)

[1] FICI values were calculated as FICI = FIC(surfactant) + FIC(antimicrobial) = [surfactant]/MIC(surfactant) + [antimicrobial]/MIC(antimicrobial). FICI ≤ 0.5 indicates synergy; 0.5 < FICI < 1.0 indicates no interaction/indifference effect; FICI > 4.0 indicates antagonism.
